# Rudimentary Forms of Disseminated Sclerosis

**Published:** 1908-01-04

**Authors:** Purves Stewart


### NOTES ON CURRENT NEUROLOGY.

### RUDIMENTARY FORMS OF DISSEMINATED SCLEROSIS.

By PURVES STEWART, M.D., F.R.C.P.

THIS disease consists of patches of sclerosis, varying in size, scattered haphazard throughout the central nervous system. The number of such areas is very variable, from many hundreds down to perhaps one or two. This being so, from the variability in the number and situation of the patches, it is difficult to conceive a " typical " example of the malady, in the sense of having a case conforming to a set clinical type. In ordinary text-book descriptions of the disease emphasis is usually laid for diagnostic purposes on the existence of such signs as nystagmus, scanning articulation, intention-tremor, exaggeration of deep reflexes and absence of sensory changes. As a matter of experience, however, for one case presenting the above symptoms we see a dozen in which any or all of them may be absent, and in which, nevertheless, the diagnosis of disseminated sclerosis is quite definite. Moreover, the course of the disease is strikingly irregular. Rarely is it steadily progressive; more usually its progress is one of successive attacks of localised weakness, followed by intervals of improvement so marked as to amount at first almost to cure. This feature of remission is so striking that a considerable proportion of cases, occurring as they so often do in young women, are at first mistaken for hysteria and treated on that assumption. Sooner or later, however, other signs make their appearance, and it becomes evident that the patient is suffering from an organic nervous malady. Let me give an illustrative case:—

A married lady, aged 40, consulted me with the following history: Ten years ago, at a time when she was run-down and worried, she had double vision, which lasted about a month and then cleared up entirely. Six years ago, at another time of family trouble, her right leg suddenly gave way when out walking. She dragged herself home with difficulty, feeling unsteady in her gait. After taking a meal the weakness entirely disappeared, and next day she was in her usual health. Five years ago she noticed that if she walked a mile or more, she staggered in her gait, the unsteadiness passing off on resting. She then had severe pain in the lower part of the spine for a fortnight, after which the power of walking steadily deteriorated. About three years ago micturition became precipitate, and this still persists. Two years ago she began to have severe attacks of giddiness, worse on walking; also subjective sensations of pins and needles in the legs on walking. A year and a half ago she had a sudden sensation of a hot glow from the hips to the toes, lasting a whole night. Eight months ago the giddiness changed in type and became most intense when she lay down, instead of on walking as previously. Also the weakness of the legs changed in character and became more localised to the left leg, which she has dragged more or less ever since. The giddiness has recently disappeared entirely. The left leg occasionally feels intensely hot. The left hand is abnormally easily tired. Her general health meanwhile has remained excellent. She has been married seventeen years and has had four healthy children, the youngest eight years ago. There is nothing to suggest syphilitic infection. The family history is excellent. On examination the patient is a healthy-looking woman, with normal speech and articulation. The optic discs are normal in appearance, the pupils and external ocular movements are normal; there is no squint, no diplopia, and no nystagmus. All the cranial nerves are normal. No anæsthesia is present. There is slight comparative weakness of the left lower face, and considerable weakness of the left lower limb at all joints. The left upper limb is neither unsteady nor perceptibly weaker than the right. The gait is broad-based, and she drags the left foot slightly, wearing out the inner side of the left sole. The supinator jerks are normal; the knee jerks are increased, especially on the left side; and there is ankle-clonus in both feet. The plantar reflexes are extensor in type. There is slight precipitancy of micturition. The cranium and vertebral column are normal.

In this case, it will be observed, there was no affection of speech or articulation, no nystagmus, and no intention-tremor. Yet, in spite of this, there can be no doubt as to the diagnosis of disseminated sclerosis. The history of the case is characteristic; its prolonged course with intermissions, the transient diplopia, the intermittent weakness first of the right leg, then of the left, gradually becoming permanent, the occasional vertigo, and the other signs, taken together, are pathognomonic of disseminated sclerosis. This is corroborated by the physical examination, which shows the existence of slight weakness of the left face and arm, and of the left leg more severely, together with increased deep reflexes and plantar reflexes of an extensor type. All these show that the affection is an organic one.

How are such cases to be treated? Unfortunately we do not yet possess any means of preventing or causing absorption of the patches of disease. All that we can do is to palliate symptoms. It is important to remember that fatigue, whether physical or mental, generally renders the patient liable to exacerbations of the disease. In every case, then, we enjoin a restful life and the avoidance of worry or over-exertion. We should discountenance violent gymnastic and other exercises which the patient has often been trying in the hope of strengthening herself. Tonics are also beneficial, not by any direct action on the disease, but by raising the power of resistance. Cure, of course, being out of the question, we must be careful in our prognosis. Perhaps the most diplomatic way of expressing our prognosis is to explain that, although the patient must not expect to throw off the complaint completely, yet with care and the avoidance of fatigue, improvement may be looked for, and that in many cases the disease remains at a standstill for a prolonged period of years.

